# Self-efficacy regarding physical activity is superior to self-assessed activity level, in long-term prediction of cardiovascular events in middle-aged men

**DOI:** 10.1186/s12889-015-2140-4

**Published:** 2015-08-25

**Authors:** Göran Bergström, Mats Börjesson, Caroline Schmidt

**Affiliations:** Wallenberg Laboratory for Cardiovascular Research, Institution of Medicine, Department of Molecular and Clinical Medicine, Sahlgrenska Academy at University of Gothenburg, Göteborg, S-413 45 Sweden; Swedish School of Sports and Health Sciences and Department of Cardiology, Karolinska University Hospital, Stockholm, Sweden

## Abstract

**Background:**

Self-efficacy has been determined to be a strong predictor of who will engage in physical activity. We aimed to evaluate the associations between self-efficacy to perform physical activity, self-reported leisure-time physical activity and cardiovascular events in a population-based cohort of middle-aged Swedish men with no previous cardiovascular disease, or treatment with cardiovascular drugs.

**Methods:**

Analyses are based on 377 men randomly selected and stratified for weight and insulin sensitivity from a population sample of 58-year-old men (*n* = 1728) and who had answered a question about their competence to perform exercise (as an assessment of physical self-efficacy). The Saltin-Grimby Physical Activity Level Scale was used to assess self-reported levels of leisure-time physical activity. Cardiovascular events were recorded during 13-years of follow-up.

**Results:**

The group with poor self-efficacy to perform physical activity had a significantly higher incidence of cardiovascular events compared with the group with good physical self-efficacy (32.1 % vs 17.1 %, *p* < 0.01). Multivariate analyses showed that poor physical self-efficacy was associated with an increased relative risk of 2.0 (95 % CI 1.2 to 3.0), of having a cardiovascular event during follow-up also after adjustments for co-variates such as waist to hip ratio, heart rate, fasting plasma glucose, serum triglycerides, systolic blood pressure, apoB/apoA-I ratio and leisure-time physical activity.

**Conclusion:**

Self-efficacy to perform physical activity was strongly and independently associated with cardiovascular events and was superior to self-assessed physical activity in predicting cardiovascular events during 13-years of follow-up in a group of middle-aged men, without known CVD or treatment with cardiovascular drugs.

## Background

Inactive lifestyles have become a predominant and pervasive feature of industrialized nations [1]. Epidemiological studies have shown that physical inactivity contributes to an early onset of cardiovascular disease (CVD) [2–6]. Conversely, regular physical activity has been shown to reduce individual cardiovascular risk factors, including blood lipids, blood pressure, and body weight, and to improve glucose metabolism [7, 8]. Accordingly, a recent meta-analysis showed that leisure-time physical activity reduces the overall risk of CVD among both men and women [9].

To assess physical activity in large populations, self-report instruments (questionnaires) are the most practical and widely used tools. The simple four-level Saltin-Grimby physical activity level scale (SGPALS), an instrument for self assessment of physical activity levels, has been shown to identify physically inactive individuals with a greater risk of elevated cardiovascular risk factors, as well as with increased risk of CVD morbidity and mortality [10, 11].

Although there is a strong consensus on the inverse relationship between physical activity and CVD, most evidence for the beneficial effect of physical activity is derived from observational studies [8, 12, 13]. It is therefore possible that these studies suffer from a positive selection bias and that people who report a high physical activity represent a healthier selection of the population who, for whatever reasons, may find it easy to exercise. Self-efficacy beliefs, as introduced by Bandura [14], are said to influence not only the courses of action pursued, but also the effort expended and endurance when facing difficulties. People with high levels of self-efficacy are more likely to pursue challenging goals, cope with pain, and persevere through setbacks, while those with low self-efficacy avoid challenges and tend to give up when confronted with obstacles [15]. Thus, poor self-efficacy regarding being physically active, may reflect a state of frailty or a less optimistic self-belief about one’s capability to perform physical activity [16]. While widely used and practical, self-assessed PA questionnaires has been shown to relatively poorly assess true PA activity and studies have shown that participants tend to report more vigorous and less sedentary time compared with objective methods [17, 18] and there is a need to identify stronger predictors than self-assessed PA, with better validity and stronger associations to outcomes.

However, self-efficacy for physical activity in itself has not previously been studied as a predictor of cardiovascular events. We hypothesized that self-estimated self-efficacy for physical activity assessed from a single question “How do you assess your competence to perform various physical activities (such as walking or jogging)” is associated with future cardiovascular events, even when adjusted for self-estimated physical activity. Hence, the aim of the present study was to evaluate the association between a subjective measure of self-efficacy for physical activity and cardiovascular events during 13-years of follow-up in a population-based cohort of healthy, middle-aged Swedish men.

## Methods

### Study population

The initial study group comprised 391 men randomly selected from a total population sample of 58-year-old men (*n* = 1728), as part of a previous study [19]. The sample size was originally calculated as the number of subjects necessary to include in order to show a significant difference in the carotid artery intima-media thickness between those in the first and fifth quintiles of fasting plasma insulin concentration (α = 0.05 and β = 0.20). The current study group has been extensively studied before [20–22], and was stratified to reflect all stages of weight as well as insulin sensitivity. However, in the present study 14 subjects had missing data on self-efficacy to perform physical activity and/or leisure-time physical activity, giving a final study group of 377 subjects with complete data. The subjects with missing data were not significantly different to the subjects, who had a complete set of data. No subjects were lost to follow-up.

All subjects were of Swedish ancestry and lived in the Gothenburg region. Exclusion criteria were CVD, clinical diabetes mellitus (fasting blood glucose ≥ 6.1 mmol/L (109.8 mg/dl) or medication) or other clinically overt disease, untreated diastolic blood pressure >100 mmHg, treatment with cardiovascular drugs (i.e. treatment of ischemic heart disease, heart failure, hypertension, hyperlipidaemia, or diabetes mellitus) or unwillingness to participate. These exclusion criteria were chosen to minimize the risk that the follow-up results are confounded by previous CVD.

The subjects received both written and oral information before giving written consent to participate. The subjects signed a consent form and the researchers saved the original, signed form and the subject got a copy of it. The Gothenburg University ethics committee approved the study (no: 89–95). The ethics committee approved this consent procedure.

### Baseline measurements

The baseline data was collected between 2nd October 1995 and 14th May 1997.

Established questionnaires were used to evaluate the medical history, including previous and current disease (s), smoking habits and alcohol consumption [19]. The total number of smoking years was multiplied by the number of cigarettes smoked daily and the product was termed 'cigarette years’ [19].

All examinations and measurements were performed at the Wallenberg Laboratory for Cardiovascular Research, Sahlgrenska University Hospital, Gothenburg, Sweden.

Weight and height were measured and body mass index (BMI) was calculated. Waist circumference was measured directly on the skin or over a single layer of light close-fitting clothing at the point between ribs and the iliac crest. Further, hip circumference was measured where the buttocks were largest (2–4 in. below the umbilicus for men). Waist to hip ratio (WHR) was calculated by dividing the waist measurement with the hip measurement. Sagittal abdominal diameter (SAD; the distance from the back to the upper abdomen, midway between the top of the pelvis and the bottom of the ribs) was measured in a supine position with subjects dressed in underwear. Blood pressure was measured twice after 5 min supine rest and the resting heart rate was recorded from a 12-lead standard-Electrocardiogram (ECG).

Venous blood samples were drawn after a fasting period of at least 6 h. Cholesterol and triglyceride levels were determined by enzymatic techniques (Thermo Fisher Scientific Oy, Vantaa, Finland). High-density lipoprotein (HDL)-cholesterol was determined after precipitation of apolipoprotein B (apoB)-containing lipoproteins and low-density lipoprotein (LDL)-cholesterol was calculated as described by Friedewald et al. [23]

Apolipoproteins (apoA-I and apoB) were measured on a Konelab 20 Auto-analyzer (Thermo Scientific, Vantaa, Finland) using a turbidimetric method.

### Assessment of physical self-efficacy on the visual analogue scale

For assessment of self-efficacy to perform physically activity (henceforth, defined as self-efficacy), we used one question from the Minor Symptom Evaluation (MSE) profile questions: “How do you assess your ability to perform various physical activities (such as walking or jogging)?” The MSE questionnaire focuses on minor symptoms affecting daily living, and the full questionnaire is a 22-item questionnaire designed to evaluate minor symptoms experienced from drug treatment [24].

A Visual Analogue Scale (VAS; a straight horizontal line 100 mm in length) was used for estimation of self-efficacy. The end points are defined in words denoting the extreme poles of the response to be measured as follows: very easy (VAS 0), indicating a high self efficacy, and very hard (VAS 100), indicating a very low self efficacy.

After the subjects had estimated their self-efficacy using the VAS, they were divided into two groups: those scoring < 50 mm and those scoring ≥ 50 mm. Scores ≥ 50 mm were defined as poor physical self-efficacy. This cut-off was chosen based on a previous study that used a VAS to evaluate the association between subjective measures of physical activity and dyslipidaemia; subjects who assessed their daily activity as active (in this case ≥50 mm) had lower LDL-cholesterol and triglyceride levels and increased HDL-cholesterol compared with the inactive group [25]. A receiver operating characteristic (ROC) curve and the area under the ROC curve (AUC) was used as a relative measure of test efficiency for events. The AUC showed to be largest at the suggested cut-off between < 50 mm and ≥ 50 mm and had an asymptotic significance of 0.039.

### Assessment of leisure-time physical activity

Self-reported level of leisure-time physical activity was also determined using the SGPALS, as previously described [10, 26]. Although the SGPALS is a four-level scale, studies have shown that less than 4 % of participants report the highest activity level [6]. In this study, only 1 % of the participants reported being physically active at the highest level. Therefore, for our study analyses, we merged the two groups with highest activity to form the vigorous physical activity group. The participants are thus divided into the following three categories:

1. Sedentary lifestyle (low physical activity): spends leisure time mostly reading, watching TV or other sedentary activities
2. Moderate physical activity: walking, cycling or other mild physical activity such as gardening, fishing or bowling at least 4 h per week
3. Vigorous physical activity: running, swimming, tennis, cross-country skiing or other exercise that leads to sweating several days per week.

The SGPALS has previously been validated against fitness levels, cardiovascular risk factors [10, 27], as well as long-term cardiovascular morbidity and mortality [11]. In addition, moderate correlations between the SGPALS and objective accelerometer data, have been reported in the Malmö Diet and Cancer study [28].

### Assessment of physical activity readiness

The study exclusion criteria ensured that all subjects had no previous cardiac or pulmonary disease or other clinically overt diseases that could affect their ability to be physically active. In addition, to account for possible reverse causations, the number of minor symptoms reported from the subjects on the study questionnaire was transferred to a Physical Activity Readiness Questionnaire (PAR-Q) and included as a covariate (pass/fail) [29]. The PAR-Q asks several questions regarding heart trouble, chest pain, high blood pressure, dizzy spells, joint problems, and other problems that may prevent subjects from participating in physical activities. Since the present cohort was previously healthy, PAR-Q questions 5 and 7 were selected to assess bone or joint problems or other reasons for not being physically active. A positive response to any question results in a failure of the PAR-Q.

### Follow-up of cardiovascular events

Cardiovascular events during 13 years of follow-up were defined as cardiovascular death, non-fatal myocardial infarction, non-fatal stroke, claudication, angina pectoris, revascularisation procedures, or hospitalisation for heart failure (only the first event was counted in each subject). The events and cause of death were collected by searching the Swedish national inpatient register (IPR) after contact with the Centre of Epidemiology at the National Board of Health and Welfare. The IPR has a high external and internal validity for CVD [30].

### Statistics

All data management and statistical analyses were conducted using PASW Statistics 18 (SPSS Inc.). Descriptive statistics were used to summarize baseline characteristics in the total sample and by physical self-efficacy and leisure-time physical activity categories. Comparisons between groups were performed using the Mann–Whitney U or Chi-square-test. Kruskal-Wallis test was used to analyze the relation between two or more categorical variables or non-normal distributed variables.

Multi-variable logistic regression analysis was used to explore the association between risk factors and cardiovascular events. The regression analyses were performed using cardiovascular events as the dependent variable and adjusting for a number of co-variates in different models (Table 3). The anthropometric and metabolic parameters were entered in the form of continuous variables, the physical self-efficacy, leisure-time physical activity and PAR parameters were entered as nominal or ordinal variables. Regression analyses were performed in three steps. In the first step, the models were adjusted for anthropometric and metabolic parameters. In the second step, the models were adjusted for PAR-Q (pass/fail) in addition to anthropometric and metabolic parameters. Finally, in the third and last step, subjects who failed the PAR-Q (*n* = 26) were excluded and the models were adjusted for anthropometric and metabolic parameters as in the first step.

## Results

### Self-efficacy to perform physical activity and level of leisure-time physical activity

**Fig. 1:**
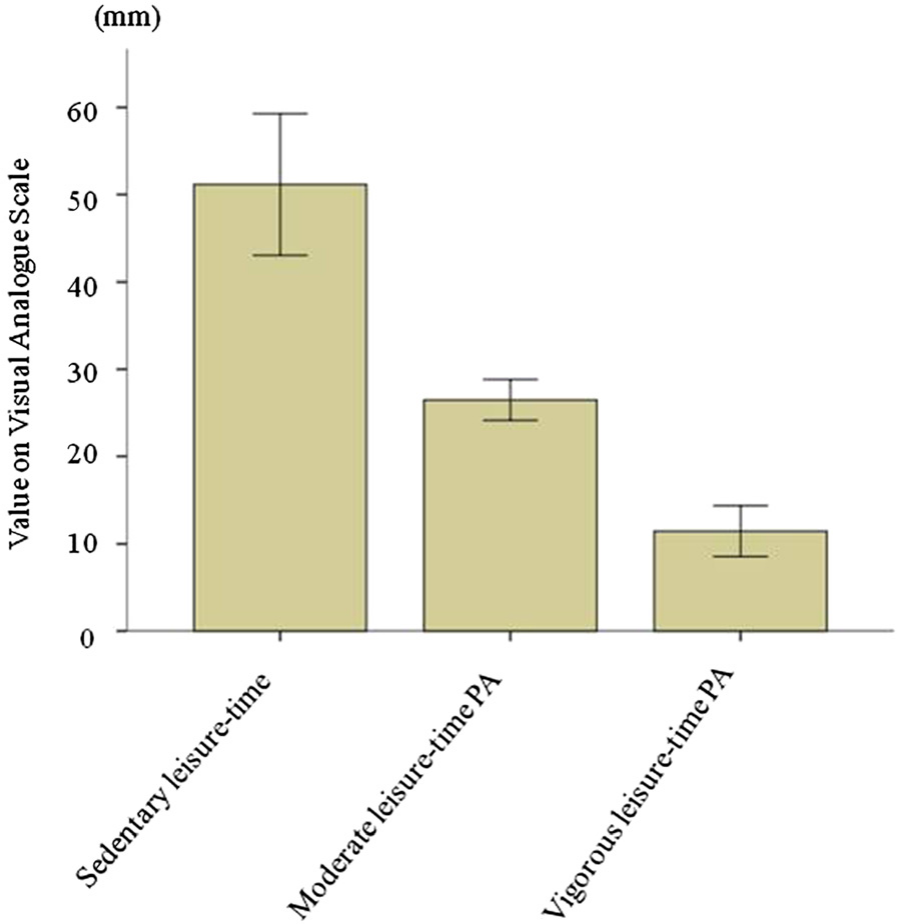
Visual Analogue Scale (VAS) values for physical self-efficacy across leisure-time physical activity groups. Increasing levels of leisure-time physical activity were associated with good physical self-efficacy i.e. lower values on the (VAS) (*p* < 0.001 for trend

The MSE question concerning self efficacy to perform physical activity was completed by 377/391 subjects (96.4 %). The majority of the population (321/377; 85.1 %) was defined as having a good self-efficacy (VAS <50 mm); the remaining 56/377 (14.9 %) were defined as having low self-efficacy (VAS ≥50 mm). Concerning leisure-time physical activity, 67 % of the population assessed their activity as moderate (260/377), 21 % as vigorous (78/377) and 10 % (39/377) as inactive. Increasing levels of leisure-time physical activity were associated with good physical self-efficacy (*p* < 0.001 for trend; Fig. 1).

Anthropometric, metabolic parameters and education length in relation to self-efficacy and leisure-time physical activity are shown in Table 1. Briefly, WHR, heart rate, fasting plasma glucose and serum triglyceride levels were higher at baseline in the group with poor self-efficacy. Furthermore, increasing levels of leisure-time physical activity were significantly associated with decreased levels of waist circumference, WHR, SAD, heart rate, triglycerides, apoB, apoB/apoA-I ratio and cigarette years.

**Table 1:** Anthropometric and metabolic characteristics of the study subjects

Characteristics	All (*n* = 377)	VAS ≥ 50 mm (*n* = 56)	VAS < 50 mm (*n* = 321)	Inactive lifestyle (*n* = 39)	Moderate PA (*n* = 260)	Vigorous PA (*n* = 78)
Weight (kg)	84.6 ± 15.0	86.8 ± 17.3	84.1 ± 14.5	87.6 ± 16.2	84.5 ± 15.1	83.4 ± 14.1
Waist circumference (cm)	96 ± 12	99 ± 14	96 ± 12	101 ± 13	96 ± 12	93 ± 11^*^
Waist-hip ratio	0.94 ± 0.07	0.97 ± 0.08^*^	0.94 ± 0.06	0.97 ± 0.08	0.95 ± 0.06	0.92 ± 0.06^***^
Sagittal abdominal diameter (cm)	22 ± 4	23 ± 4	22 ± 3	23 ± 4	22 ± 4	20 ± 3^*^
Blood pressure (mmHg)						
Systolic	129 ± 17	132 ± 17	129 ± 17	130 ± 18	130 ± 18	126 ± 13
Diastolic	77 ± 10	77 ± 11	77 ± 10	78 ± 11	77 ± 10	76 ± 8
Heart rate (bpm)	64 ± 10	67 ± 10^*^	64 ± 10	69 ± 10	64 ± 10	60 ± 8^***^
Plasma glucose (mmol/L)	5.5 ± 1.3	5.9 ± 2.0^*^	5.4 ± 1.1	5.9 ± 2.0	5.9 ± 2.0	5.9 ± 2.0
Serum cholesterol (mmol/L)						
Total	6.01 ± 1.11	6.20 ± 1.22	5.96 ± 1.07	6.18 ± 1.24	6.02 ± 1.15	5.91 ± 0.85
LDL	4.02 ± 0.93	4.17 ± 1.08	4.02 ± 0.93	4.10 ± 1.01	4.08 ± 1.00	3.94 ± 0.79
HDL	1.27 ± 0.36	1.27 ± 0.41	1.27 ± 0.36	1.20 ± 0.32	1.26 ± 0.36	1.36 ± 0.43
Triglycerides^a^	1.32 ± 1.06	1.56 ± 0.95^*^	1.32 ± 1.06	1.67 ± 1.82	1.36 ± 0.83	1.23 ± 1.19^*^
Apolipoproteins (g/L)						
ApoB	1.20 ± 0.27	1.27 ± 0.29	1.20 ± 0.27	1.27 ± 0.26	1.22 ± 0.29	1.15 ± 0.23^*^
ApoA-I	1.42 ± 0.22	1.42 ± 0.27	1.42 ± 0.22	1.35 ± 0.20	1.42 ± 0.22	1.46 ± 0.26
ApoB/apoA-I ratio	0.87 ± 0.24	0.93 ± 0.28	0.87 ± 0.24	0.97 ± 0.26	0.88 ± 0.24	0.82 ± 0.25^*^
Smoking (yes) ^b^ n, %	89, 23.6	11, 19.6	78, 24.3	11, 28.2	68, 26.2	10, 12.8^f^
Cigarette years	333 ± 419	358 ± 391	333 ± 419	499 ± 498	342 ± 412	216 ± 334^***^
Alcohol intake (g/day)	17.4 ± 15.8	16.0 ± 14.3	17.4 ± 15.8	19.5 ± 16.4	17.1 ± 16.0	15.2 ± 11.3
PAR^c^ failure, %	26, 6.9	11, 19.6^***^	15, 4.7	4, 10.3	17, 7.0	5, 6.8
Lenght of education (years)	11.8 ± 3.8	12.1 ± 4.0	11.8 ± 3.8	11.4 ± 3.9	11.6 ± 3.7	12.6 ± 4.1

^a^ Geometric mean

^b^ Pearson Chi-square

^*^ <0.05, ^***^ <0.001 compared to VAS <50 mm; ^*^ <0.05, ^***^ < 0.001 for trend

The number of minor symptoms that may affect physical activity readiness (as assessed using the PAR-Q) was significantly higher in the group with poor self-efficacy compared with the group with good self-efficacy (Table 1).

### Cardiovascular events

In the 13-year follow-up, 73 fatal and non-fatal cardiovascular events were recorded (Table 2). Of these, the main events were stroke and myocardial infarction (Table 2).

**Table 2:** Cardiovascular events according to physical self-efficacy and leisure-time physical activity

	VAS ≥ 50 mm (*n* = 56)	VAS < 50 mm (*n* = 321)	Inactive lifestyle (*n* = 39)	Moderate PA (*n* = 260)	Vigorous PA (*n* = 78)
Myocardial infarction n, (%)	5 (8.9)	13 (4.0)	4 (10.3)	12 (4.6)	2 (2.6)
Stroke n, (%)	7 (12.5)	27 (8.4)	5 (12.8)	22 (8.5)	7 (9.0)
Claudication n, (%)	0 (0)	2 (0.6)	0 (0)	2 (0.8)	0 (0)
Revascularization n, (%)	2 (3.6)	9 (2.8)	1 (3)	5 (1.9)	5 (6.4)
Heart failure n, (%)	4 (7.1)	2 (0.6)	0 (0)	6 (2.3)	0 (0)
Angina n, (%)	0 (0)	2 (0.6)	0 (0)	2 (0.8)	0 (0)
Total n, (%)	18 (32.1)	55 (17.1)^**^	10 (25.6)	49 (18.8)	14 (17.9)

Pearson Chi-square; ^**^ <0.01

There was no difference in the incidence of cardiovascular events between the groups who were physically inactive at leisure time at baseline, and those with higher levels of leisure-time physical activity (Table 2). In contrast, the group with poor self-efficacy at baseline had a significantly higher incidence of cardiovascular events compared with the group with good self-efficacy (Table 2).

The group that had a cardiovascular event during follow up, was observed to have an increased blood pressure and apoB/apoA-I ratio compared to the group which had no event (134 ± 19 vs. 128 ± 17, p = 0.009 and 0.94 ± 0.27 vs. 0.86 ± 0.24, p = 0.017, respectively). No other differences were observed.

### Multivariate regression analysis

Self-efficacy to perform physical activity showed to be an independent and strong (significant) predictor for cardiovascular events also after adjustment for cardiovascular risk factors. Additionally, when the models were controlled for activity level, according to SGPALS, self-efficacy remained an independent predictor of CV events. When excluding the subjects who failed the PAR-Q from the regression analysis, self-efficacy was still a strong significant predictor for cardiovascular events with maintained risk magnitude (Table 3).

**Table 3:** Multivariate regression analysis with cardiovascular events as dependent variable

		Step 1 (*n* = 377)			Step 2 (*n* = 377)			Step 3 (*n* = 351)		
Model	Variables included	Risk Ratio	95 % CI	*p*-value	Risk Ratio	95 % CI	*p*-value	Risk ratio	95 % CI	*p*-value
1	Physical self-efficacy	1.9	1.2 to 2.8	0.010	1.9	1.2 to 2.8	0.012	1.9	1.2 to 2.9	0.011
2	Physical self-efficacy + WHR, heart rate, fasting plasma glucose, serum triglycerides	1.9	1.2 to 2.8	0.015	1.9	1.2 to 2.8	0.016	1.9	1.2 to 3.0	0.016
3	Physical self-efficacy + WHR, heart rate, fasting plasma glucose, serum triglycerides + leisure-time PA	1.9	1.2 to 2.9	0.014	1.9	1.2 to 3.0	0.015	2.0	1.2 to 3.1	0.018
4	Physical self-efficacy + SPB and apoB/apoA-I ratio	1.9	1.9 to 2.9	0.014	1.9	1.2 to 2.9	0.014	2.0	1.2 to 3.0	0.010
5	Physical self-efficacy + SPB and apoB/apoA-I ratio + leisure-time PA	1.9	1.2 to 2.9	0.014	1.9	1.2 to 3.2	0.015	2.0	1.2 to 3.1	0.015
6	Physical self-efficacy + WHR, heart rate, fasting plasma glucose, serum triglycerides, SPB, apoB/apoA-I ratio and leisure-time PA	2.0	1.2 to 3.0	0.010	2.0	1.2 to 3.1	0.010	2.1	1.2 to 3.2	0.010

CI, Confidence interval

Step 1- the models are adjusted for anthropometric and metabolic parameters; Step2- the models are adjusted for PAR-Q (pass/fail) and for anthropometric and metabolic parameters; Step 3- subjects who failed the PAR-Q were excluded (*n* = 26) and the models were adjusted for anthropometric and metabolic parameters as in Step 1

## Discussion

The main finding of the present study of middle-aged men with no known cardiovascular disease at baseline was that the incidence of cardiovascular events during 13 years of follow-up was significantly higher in men with poor self-efficacy for physical activity at baseline, compared to men with high self-efficacy. Indeed, the increased risk was significant also after adjustment for classical cardiovascular risk factors, such as systolic blood pressure and apoB/apoA-I ratio. In contrast, there was no significant difference in the incidence of cardiovascular events between the groups when the study subjects were divided according to their self-reported level of leisure-time physical activity, at baseline.

Interestingly, in the present study, when both self-efficacy and leisure-time physical activity were included in the regression models, only self-efficacy to perform physical activity was shown to be a significant and independent predictor for cardiovascular events. Although we observed a co-variation between self-efficacy and leisure-time physical activity, the correlation was only moderate. The two variables could hold different information or alternatively they may both reflect the level of PA although differently. Efficacy expectations influence the behaviour through a number of processes. Physical activity is composed of challenging tasks and efficacy beliefs seem to be important motivational regulators of this sort of activity [31], and a study has shown that participants with high self-efficacy reported significantly greater positive well-being and less psychological distress and fatigue during exercise and after, despite exercising at the same level of intensity as participants with low self-efficacy [32]. Also, studies comparing self-reported PA and objectively measured PA have shown large differences, where subjects overestimate their PA compared to objectively measured PA, especially with regard to vigorous intensity and particularly among overweight subjects. Respondents in general may experience difficulty assessing the intensity of an activity and overweight subjects may rate an activity as vigorous more easily than normal weight subjects [17].

Our data differs somewhat from other previous studies. For example, a meta-analysis with data from 26 studies, including over 510,000 individuals, showed that a high level of leisure-time physical activity provided significant protection against coronary heart disease [33]. In our study, we could not show any association between leisure-time PA level and cardiovascular events at follow-up. Although the used scale for assessment of PA-level, has been shown to discriminate between risk factors, individual fitness levels and cardiovascular morbidity in earlier studies, self assessment of PA is still inferior, in estimating the true PA-level of the individual, as compared to more objective assessments of PA, such as measurement of VO2-max and/or accelerometer data [34–36]. As a result of the present study, one might speculate that the subject´s own assessment of his/her self-efficacy for being physically active may estimate a more true level of PA of the individual, better than the PA-level assessed by direct questions.

Not surprisingly, cardiovascular events were associated with classical risk factors such as blood pressure and apoB/apoA-I ratio in this cohort, as have been shown in many previous studies. Observational and interventional studies have shown that blood pressure levels are strongly and directly related to the relative risks of stroke and heart disease [37]. Furthermore, both the INTERHEART and the INTERSTROKE studies showed apoB/apoA-I ratio is a strong predictive variable for myocardial infarction as well as stroke [38, 39].

If self-efficacy for physical activity reflects the level of PA of the patient, we would expect an association with self-efficacy to different cardiovascular markers, by which PA traditionally conveys its positive effects on CVD. However, no uni-variate association between self-efficacy, blood pressure and apoB/apoA-I ratio, was observed in the present study. In contrast, poor self-efficacy was associated with unfavourable alterations in metabolic variables such as increased WHR, increased fasting plasma glucose and serum triglyceride levels.

An interesting alternative explanation is that self efficacy to perform physical activity, may instead be a proxy for identifying a selection of individuals having other advantages, potentially making them less likely to develop CVD. This may be a genetic predisposition, a positive mental state or the effect of previous environmental exposure that coincides or results in a lower risk for CVD. It may even be that self-efficacy for physical activity is an individual positive quality that has a stronger predictive value for cardiovascular disease than individual activity level in itself. The findings of the present study may have important clinical implications. Indeed, it is tantalising to speculate that a single question reflecting self-efficacy may be a clinical instrument for risk prediction.

Importantly, we found a difference in physical readiness between the groups that reported poor versus good self-efficacy. However, when responses to the PAR-Q were included as a covariate in the multivariate models, the results were unchanged. Furthermore, when the subjects who failed the PAR-Q were excluded from the cohort and the multivariate models were adjusted for anthropometric and metabolic parameters and leisure-time physical activity, the association between self-efficacy and cardiovascular events was still maintained.

There are some limitations to the present study that need to be addressed. VAS provides a subjective rather than objective measure of the clinical phenomenon and is thus subject to potentially higher error rates [25]. Further, the MSE question used for assessing self-efficacy cannot distinguish if a subject has different self-efficacy for different activities (e.g. would they be happy to run but not to cycle?). This should be addressed in future studies. Another limitation of the study is external validity, i.e. that this cohort only included men of Swedish ancestry within a limited age category, and the results found in the present study may not be extended to other age groups, women or other ethnicity groups. The reason that we could not observe a difference between leisure-time physical activity levels and cardiovascular events in the present study could be due to not having sufficient power. A further limitation is that medical data concerning depression was not available in this study and low-self-efficacy expectancies are an important feature of depression [14], this may have affected physical self-efficacy. However, all subjects were clinically healthy eliminating sever forms of depression.

Nevertheless, one strength of our study is that we investigate a well-characterized cohort of men of the same age of Swedish ancestry, living in the Gothenburg area, which controls for variation in age, sex and ethnicity. Further, the included subjects were initially free of CVD, clinical diabetes mellitus or other clinically overt disease, treatment with cardiovascular drugs, such as treatment of ischemic heart disease, heart failure, hypertension, hyperlipidaemia, diabetes mellitus, which might otherwise have confounded the interpretation of results.

## Conclusion

In summary, we showed that self-efficacy to perform various physical activities, such as walking or jogging, self-assessed using a VAS, was strongly and independently associated with cardiovascular events during 13-years of follow-up in a group of middle-aged men without a history of CVD or diabetes and who were not taking cardiovascular drugs. Furthermore, we showed that subjectively judged poor self-efficacy was superior to self-assessed physical activity in predicting cardiovascular events. Self-efficacy to perform physical activity may be a better predictor of CVD events, partly by better assessing the true level of PA of the individual, and possibly by identifying other, as yet unknown, risk factors. It remains, however, to be investigated whether a single question assessed on a VAS can be used as a rapid screening tool for the prediction of future cardiovascular events in the clinical setting.
